# Supplementary material for: Rumen Fermentation, Digestive Enzyme Activity, and Bacteria Composition between Pre-Weaning and Post-Weaning Dairy Calves
Source: Animals (Basel). 2021 Aug 28;11(9):2527. doi: 10.3390/ani11092527 (PMC8467862; doi:10.3390/ani11092527)
Supplement: Supplementary file 1 [file animals-11-02527-s001.zip › animals-1320837-supplementary.pdf]

Table S1. Nutrient composition of starter and oat grass (Dry matter basis)

Nutrient level [1]	Starter [2]	Oat grass
DM	86.94	91.79
CP	20.20	6.28
EE	4.38	1.73
Ash	5.43	7.95
NDF	11.62	54.34
ADF	4.88	30.41
Starch	33.95	1.23
Ca	0.89	0.26
P	0.50	0.20

[1] DM: dry matter, CP: crude protein, EE: ether extract, NDF: neutral detergent fiber, ADF: acid detergent fiber, Ca: calcium, P: phosphorus.

[2] Starter composition: 47.65% Cornmeal, 18.47% Soybean meal, 20.33% Extruded soybean, 5.26% Corn gluten meal, 2.74% Sugarcane molasses, 5.55% Mineral-vitamin premix.

Table S2. The enzymes and their corresponding reacting substrate and wavelength

Enzyme	React substrate	Be tested product	Wavelength/nm
dehydrogenase	nitrogen tetrazolium salt (CN_4H_2)	formazan (CN_4H_4)	460
urease	Urea (CH_4N_2O)	ammonia nitrate (NH_3-N)	578
protease	Casein ($C_{81}H_{125}N_{22}O_{39}P$)	Tyrosine ($C_9H_{11}NO_3$)	680
lipase	p-nitrophenol butyrate ($C_{10}H_{11}NO_4$)	p-nitrophenol ($C_6H_5NO_2$)	405
glucosidase	p-nitrophenyl-β-D-glucopyranoside ($C_{12}H_{15}NO_8$)	p-nitrophenol ($C_6H_5NO_2$)	405
carboxymethyl cellulase	sodium carboxymethylcellulose ($C_6H_7O_2(OH)_2OCH_2COONa$)	reducing sugars	540
cellobiohydrolase	microcrystalline cellulose ($(C_6H_{10}O_5)n$)	reducing sugars	540
amylase	Starch ($(C_6H_{10}O_5)n$)	reducing sugars	540
xylanase	Xylan ($(C_5H_8O_4)n$)	reducing sugars	540

Under alkaline conditions, the produced reducing sugars can react with 3,5-Dinitrosalicylic acid ($C_6H_5NO_3$) reacts to produce a brown-red substance, which can be measured under 540nm wavelength.

All the chemical reagents were produced from the company of Suzhou Grace Biotechnology Co., Ltd, Jiangsu, China.
